# Electrical Curing of Metakaolin- and GBFS-Based Geopolymers: A Sustainable Technology Aligned with the European Green Deal

**DOI:** 10.3390/ma18204811

**Published:** 2025-10-21

**Authors:** Yusuf Gokcegoz, Mucteba Uysal, Orhan Canpolat, Oktay Arikan, Hasan Dilbas, Beyza Aygun

**Affiliations:** 1 Civil Engineering Department, Civil Engineering Faculty, Yildiz Technical University, Istanbul 34220, Türkiye; mucteba@yildiz.edu.tr (M.U.); canpolat@yildiz.edu.tr (O.C.); 2 Electrical Engineering Department, Yildiz Technical University, Istanbul 34220, Türkiye; oarikan@yildiz.edu.tr; 3 Civil Engineering Department, Van Yuzuncu Yil University, Van 65080, Türkiye; hasandilbas@yyu.edu.tr; 4 Construction Technology Program, Vocational School, Istanbul Esenyurt University, Istanbul 34513, Türkiye; beyzaaygun@esenyurt.edu.tr

**Keywords:** carbon black, carbon fiber, electrical conductivity, erosion wire, multi-criteria decision support

## Abstract

On-site curing of metakaolin (MK)- and granulated blast furnace slag (GBFS)-based geopolymer mortars remains a major bottleneck compared to thermal treatment for early strength development, and electrical curing is proposed here as a highly scalable and energy-efficient alternative technology. Geopolymer mortars with 0–100% MK/GBFS binder ratios were activated using sodium silicate (SS) and sodium hydroxide (SH) solutions of the following molarities: 6, 8, 10, 12, and 14 M. Steel fiber (SF), carbon fiber (CF), waste erosion wire (EW), and carbon black (CB) microfiller were incorporated to enhance the electro-conductive efficiency of the geopolymer matrix. Specimens were subjected to electrical curing under 10 V and 20 V AC and were compared with benchmarking under ambient conditions of 23 °C and thermal conditions of 70 °C. The findings established that the incorporation of fibers substantially boosted the level of conductivity and mechanical performance, with 28-day compressive strengths of up to 88.30 MPa (0.50% EW, 20 V) and flexural strengths of up to 22.24 MPa (0.50% CF, 7 days), exceeding the results of conventional curing in various instances. Microstructural studies based on well-bonded geopolymer gels with fibers indicated uniform geopolymerization through electrical curing without deleterious fiber–matrix interactions. A multi-criteria decision support approach (the HD method) based on 273 parameters established 0.50% CF, 0.75% SF, 0.75% EW, and 1.00% CB as the group-wise optima and chose 0.75% EW as the single-best performing combination. The findings confirm that electrical curing is a low-carbon, cost-effective, and field-adjustable curing technology with the potential to achieve target strength ratings, in line with the European Green Deal’s climate-neutral building material goals.

## 1. Introduction

Low- and net-zero-carbon technologies are shaping the global economy and promoting growth with low costs and energy requirements. This topic is currently a priority for decision-makers and researchers [1]. The European Union’s 100 smart and climate-neutral cities are a highly impacted plan in Horizon 2030. This plan designs future cities, especially buildings. According to Horizon 2030, investigators/designers are encouraged to submit their research to shape the future. Low-carbon technologies (i.e., in terms of building materials such as geopolymers) have gained importance nowadays, and this subject contributes to recent advances and future projects [1,2].

Portland cement (PC) is the most commonly used binder material in the construction industry. Therefore, shaping the future using low-carbon technologies and materials begins with the elimination of cement and its replacement with environmentally friendly alternatives. The cement industry consumes about 3.8% of total global energy [3]. Alternative binders in concrete production have begun to be investigated worldwide. For this purpose, geopolymers have been intensively researched for over 40 years thanks to their environmental benefits, energy savings between 40% and 80%, economic benefits, and superior mechanical durability properties. These materials play a crucial role in reducing the ecological impact of PC and in utilizing industrial waste, making them valuable new engineering materials [4,5,6,7].

Metakaolin (MK) is a clay mineral formed at high temperatures (550–800 °C). Therefore, MK is used for its easy availability and production [8]. It is chosen as a repair material owing to its fast compressive strength of up to 100 MPa for 28 days, excellent thermal durability, and good reaction in alkali solutions [7,9,10]. GBFS is a by-product of pig iron production in blast furnaces, formed from limestone, iron ore, and coke [8]. It has been studied that many pozzolanic materials are the main component in the production of geopolymers, and according to the results, metakaolin-slag-based geopolymers are the best-produced composites in terms of their acceptable mechanical and durability performance, as well as ecological performance. Adding 20% GBFS to MK-based geopolymers significantly reduces setting time while enhancing flowability and compressive strength [11].

Generally, geopolymers are cured using traditional oven heating and ambient curing methods. Reaction temperature is a critical factor in geopolymer production. The reaction rate is very low at ambient temperature, and curing is unlikely due to delayed settings [12,13]. However, the reaction rate increases significantly while the curing temperatures increase from 40 °C to 85 °C. It has been declared that the early strength gain when preparing with different precursors can achieve a strength range of 70–100 MPa for 28-day compressive strength [11,14]. The chemical properties of aluminosilicate materials used in geopolymer mortars significantly affect their conductivity. Studies state that the conductivity of the samples prepared with fly ash and metakaolin is good, and metakaolin shows much better thermoelectric behavior. Electrical curing offers faster strength development, lower operational costs, and improved quality control and prevents freezing of fresh concrete when additives are used at low voltages [15,16,17,18,19]. It has been stated that increasing the NaOH concentration and water content improves both physical and electrical properties [20,21]. Additionally, the workability of geopolymers is the main criterion during the production of geopolymers, and high workability can enhance the compactness of the medium, lowering air bubbles [1]. Significant problems will cause difficulty compacting and a weak final structure unless necessary precautions are taken. Kovarik et al. [22] researched that electricity boosting as a poly-alumino-sialate curing process could help investigate clay residues activated with potassium silicate through differential scanning calorimetry. They concluded that the mechanical properties showed no differences in flexural strength between electrically and usually cured samples.

CF is widely used in concrete for its affordability, high modulus of elasticity, tensile strength, and toughness. With CF, the samples’ electrical resistance is lowered thanks to a descending trend with increasing stress. Payakaniti et al. [6] and Dehghanpour et al. [23] have stated that CF addition increases conductivity and compressive strength. Vaidya and Allouche [24] have used CF to enhance the electrical conductivity of geopolymers with the aim of health monitoring. SF is one of the most used fibers in mortars and concretes to increase toughness, flexural strength, and resistance to high temperature, impact, and dynamic loads and avoid cracking. SF has high tensile strength, modulus of elasticity, and strain properties compared with those of other fibers [25,26]. CB, obtained through the pyrolysis method from waste tires, is one of the most used materials in cementitious composites to increase conductivity and mechanical properties. CB is favored for its high conductivity, low cost, and nanoporous agglomerate structure. Wen and Chung [27] and Monteiro et al. [28] have suggested that the optimum ratio of CB addition is 4% replacement of cement. To learn the heating capacity, Cai et al. [12] use CB and SF together in a fly-ash-based geopolymer. They reported that self-electrical heating could increase economic efficiency thanks to its lower energy and higher curing capacity. EW obtained through cutting using electro-thermal energy has been commonly used in metalworking industries. Ipek [29] found through study that EW increases the mechanical properties of the concrete and assures that it is more economical than steel fibers. Dehghanpour and Yilmaz [30] selected 20 wt. % CB and 0.5–1 vol. % CF to search for an economical evaluation of conductive concrete, and 95–120 V voltages were selected for slab samples.

Although geopolymer systems have gained wider attention in the past two decades, the research trends still do not effectively deal with the multi-dimensional issues associated with the use of such materials in realistic field settings, especially in cold or energy-starved conditions. Thermal curing methods for fast-tracking geopolymerization only remain effective with controlled environments and high-input energies. Likewise, since the response of geopolymer systems has mainly been examined in terms of isolated mechanics or durability metrics, little attention has been given to the highly coupled nature of chemical reactions, microstructural compaction, transient phenomena of heat and mass transfer, and electromechanical behaviors. Also, for the majority of geopolymeric precursor blends and activator mixtures, stepwise variations result in the absence of holistic, decision-oriented assessment methods that can be used to predict the suitable blend selection for particular operational and environmental constraints, forming, in that regard, a fragmented database that inhibits scientific advancements and industrial applications of these materials. Thus, broad and deep epistemological, methodological, and technological voids remain at the interface of innovative curing technologies, multi-criteria performance evaluation, and strategic material engineering, for which systematic methods barely exist. Filling such voids is essential not only for enhancing our concomitant fundamental understanding but also for bridging, effectively and efficiently, innovation established at the laboratory scale with reality on the ground. To this end, this present work posits an innovative combination of low-voltage electrical consolidation utilizing fiber-based conductivity enhancement to facilitate fast and efficient matrix consolidation and subjecting the multi-dimensional integrated performance analysis to a holistic, data-informed evaluation paradigm. By bridging microstructural evolutions and macroscopic properties and decision support outputs, the present study presents, for the first time, a new methodological and technological framework that is beyond its prior parameter-driven counterparts in traditional studies. Beyond being the first-order academic research basis reference, in the future, this work will also present directly usable information for practicing engineers and decision-makers in their quest for sustainable, high-performance solutions in place of PC-based systems in the wake of maturing environmental regulations and policy regimes.

In this context, an alternative methodological and technological framework was proposed by applying direct electrical curing with conductive fibers in order to overcome the above-mentioned shortcomings. In the first stage of the experimental study, the physical and mechanical properties of MK- and GBFS-based geopolymers are investigated. In the second stage, fibers are added at different ratios to improve electrical conductivity and thus the properties of geopolymer mortars. During electrical curing, the current passing through the samples was monitored using ammeters until the final setting. Microstructural analyses and observations, such as Scanning Electron Microscope (SEM), Energy-Dispersive X-Ray Spectroscopy (EDS), X-ray diffraction (XRD), and Fourier Transform Infrared Spectroscopy (FTIR) analyses, were conducted to characterize the microstructure of the reaction products. In the last stage, an HD method is applied using parameters such as the physical properties, the mechanical properties, the chemical properties, and the current value. In contrast, electrical curing is used to evaluate the best geopolymer objectively and holistically. EU’s Horizon 2030 will embrace 100 smart and climate-neutral cities, and using electrical curing holds significant potential for net-zero materials. This topic aims to contribute to the European Green Deal and the green material subject for climate-neutral cities. Besides being a primary scholarly reference, it also offers workable protocols for its integration based on sensor networks and intelligent monitoring systems, situating geopolymer composites within the larger smart infrastructure and smart city framework, for which real-time monitoring of performance, adaptational control, and energy efficiency are key aspects of sustainable building.

## 2. Materials and Methods

### 2.1. Material Characterization

**Figure 1:**
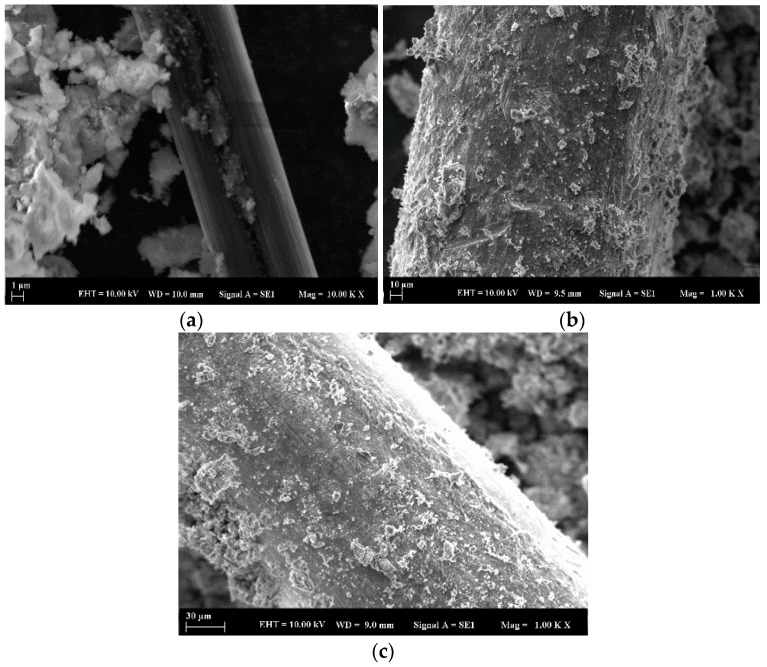
SEM images of the fibers: (**a**) CF, (**b**) EW, and (**c**) SF.

Kaolin Industrial Mining Company (Türkiye) supplied the MK. MK has a bulk density of 2520 kg/m^3^, a surface area of 8600 m^2^/kg, and a SiO_2_ + Al_2_O_3_ + Fe_2_O_3_ ratio of 97.18%, which means high pozzolanic activity. MK is fine-grained, as the amount remaining on a 45 µm sieve is 0.70%. MK was selected as the Si-Al source material. GBFS has a bulk density, a 2910 kg/m^3^ surface area of 410 m^2^/kg, and a SiO_2_ + Al_2_O_3_ + CaO ratio of 88.96%. GBFS is fine-grained, as the amount remaining on a 45 µm sieve is 1.40%. GBFS was used as the Si-Ca source material. SS and SH are commonly used alkaline reactant solutions, and Na-based materials are opted for in geopolymer production because they are more economical and beneficial [11,14]. Torgal et al. [31] have suggested that the most suitable alkali activators are made of hydroxides and soluble silicas. The sodium silicate (Na_2_SiO_3_) was a three-module form (Ms: SiO_2_/Na_2_O ≈ 3) consisting of 8% Na_2_O, 27% SiO_2,_ and 65% H_2_O. Fine aggregate smaller than 2 mm was selected from naturally siliceous river sand to prepare the geopolymer. This sand has a unit weight of 1352 kg/m^3^, 99% purity, a bulk density of 2563 kg/m^3^, and a saturated dry surface. Sieve analysis of the sand is given in Table 1. Additionally, the geometric and electrical properties of the fibers and their SEM analyses are presented in Table 2 and Figure 1, respectively.

### 2.2. Experimental Procedure

In the study, MK and GBFS were selected as binders in the production of the geopolymer and were mixed at varying proportions, such as 0, 25, 50, 75, and 100% by weight. Huseien et al. [10] examined MK-GBFS (0–15%)-based geopolymers and provided insightful explanations about their early strength properties. SH is prepared at five molarities: 6, 8, 10, 12, and 14 M. In the literature, many studies have shown that NaOH concentrations from 4 M to 14 M are useful for geopolymers [11]. Jaya et al. [32] studied geopolymers with the same molarities and suggested that 10 M was ideal for MK-based geopolymers. In the alkali activator solution, the SS-to-SH ratio was set as 2.0. Previous studies have shown that using a ratio of 2.5 led to impressive strength [30]. It has been mentioned that an increase in the ratio of Na_2_SiO_3_/NaOH up to about 1.5–2.5 improves the compressive strength, but a higher ratio decreases the strength [11,33,34]. This may occur because of an excessive SS content that slows the geopolymerization process. To determine the physical properties, tests for bulk density, water absorption, and porosity were carried out at 28 days using the standard of ASTM C642 [35]. Firstly, the dry ingredients were mixed for 30 s, and then the activators were added. When 3 min had passed, the sand was added. After a 6 min mixing process using a mechanical blender, fibers were added, and it was mixed for an additional 90 s. For the compressive strength tests, 50 × 50 × 50 mm cubic samples were used, according to the standard of ASTM C109 [36]. For flexural strength tests, 40 × 40 × 160 mm prismatic samples were used, according to the standard of ASTM C348 [37]. Cylindrical samples for splitting the tensile strength at a size of 100 × 200 mm were cast and tested at 28 days according to the standard of ASTM C496 [38].

**Figure 2:**
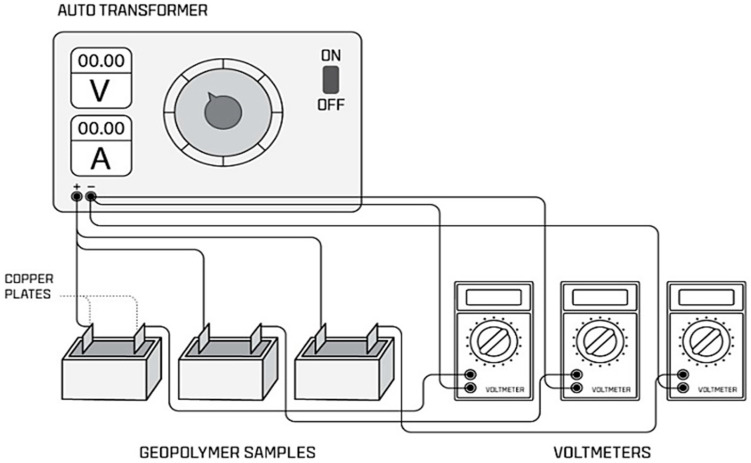
Experimental system of electrical curing and test setup.

Electrical energy was implemented in the samples using copper plates, which were suitable for the sample shape. Wooden molds were used for electrical curing implementation. Electrical curing was applied to one sample group of a series for 24 h at 10 V and the second group at 20 V, the third one was applied with ambient curing at 23 °C, and the last one was thermally cured at 70 °C for 24 h in an oven. The samples used for electrical curing were demolded after 24 h. Previous studies have proposed that the curing temperature can range between 60 °C and 95 °C [14]. At this stage, 30 V voltages were tried in the first experiments, but the results obtained were at an unsatisfactory level and were found to be low compared to those at 10 V and 20 V. When the voltage applied was high, the samples warmed too fast. Therefore, this caused the mixture to dry before geopolymerization occurred. Therefore, 30 V electrical curing was not used. The experimental system for electrical curing was designed and applied, and the samples were tested, as shown in Figure 2.

To appropriately characterize the electrothermal environment in electrical curing, the electrical behavior of the specimens was continually measured for the first curing phase. The maximum current, in turn, usually achieved after voltage application, was systematically recorded for each composition and curing state. This measurement was chosen as the qualitative measure of the curing intensity due to its representation of the moment at which, and only exactly when, the conductive network in the fresh geopolymer matrix is fully established and current conduction is stabilized. The peak current emphasizes the synergies of ionic conductivity in the pore solution, liquid phase connectivity, and saturation level and the formed network being penetrated by the effects of the in situ conductive fibers (EW, SF, CF). When the activator solution penetrates the precursor solid structure, Na^+^ and OH^−^ ions act as charge carriers, and the electric field applied enhances their conveyance through the pore network’s connectivities. Meanwhile, metallic fibers provide preferred current paths, which reduce the overall electrical resistance. This cooperation triggers local Joule heating, which triggers dissolution–polycondensation reactions before those in thermally cured systems and triggers fast setting and matrix compacting. This facilitates repetition of the peak current for a given mixture–voltage combination, which is the moment of critical current stabilization and the highest power input. While continuous temperature–time histories and curves of power consumption were not monitored in this process due to the non-existence of embedded thermocouples and power meters, the curing process was applied diligently for uniformity in all mixtures. Each specimen was cured at a constant voltage, constant ambient conditions, and a normalized geometry to trigger a uniform current density and comparable thermal boundary conditions. Under these conditions, the peak current was employed as an effective surrogate for the effective intensity of the electrocuring, just like the peak temperature was used to characterize the thermal curing regimes. This approach is backed up by physical insights into conductive heat generation in porous cementitious systems, in which the max current, along with the maximum instantaneous power (P = V × I), is directly related to the peak heating rate in the system. Systematic measurement of the voltage, current, curing time, and mixing parameters made it possible to make controlled comparisons of batches with varying binder compositions, activator molarity, and fiber content and thus to make meaningful measurements of the effects these parameters had on the electrothermal environment and, in turn, the mechanical and microstructural maturity of the geopolymers. Direct temperature profiling supplies an extra spatial and temporal resolution, and the adopted protocol supplies a technically robust and internally consistent basis for a comparative analysis of the electrocuring behavior in multiple mix designs.

While the mixes were prepared, different ratios of binder/activator (B/A) were tried. Three ratios of B/A were selected: 1.00, 1.25, and 1.40. At this stage, flow table tests were applied. For a 1.40 B/A ratio, the flow result for the control sample was 16 mm, while the fiber-added samples achieved 14 mm. For a 1.00 B/A ratio, the result was 20 mm, while fiber-added ones are 18 mm. Lower and higher ratios were examined, but they were not opted for because it was seen that the higher the ratio, the lower the strength. When the ratio was low, the mixture became too clumpy to be mixed. Thus, the workability of the mixes was lost, and molds could not be cast for investigation. Pinto [39] and Heah et al. [13] explained that this fact is due to the viscous nature of liquid Na_2_SiO_3_, leading to a sticky geopolymer paste. Aziz et al. [40] researched the B/A ratio and found nearly the same ratios. A low ratio (1.0) decreased the quantity of SiO_2_, Al_2_O_3,_ and CaO, while these were the main contents in geopolymerization.

Table 3 shows the mixing proportions of the prepared geopolymer mortars for five NaOH molarities and three B/A ratios.

In the second stage, fibers were added at the selected proportion as 0.25, 0.50, and 0.75% by weight for SF, CF, and EW because it was declared that the performance of the geopolymer composites improved enormously with the addition of fibers at a volume of 0–1.2% in previous studies [26,41]. Besides this, CB was also added autonomously as a conductive microfiller at 1, 2, and 3% weight for better electrical conductivity and densification of the matrix for the composites. Then, mechanical and physical tests were performed to understand the effect of fibers on the electrical conductivity and strength. Cube samples of 50 × 50 × 50 mm were cast and tested on the 28th day, according to ASTM C642 [31]. Compared to the control mix, adding fibers did not significantly change the density of the manufactured geopolymer mortars. Adding CF, SF, and EW slightly increased the densities of the fiber-added geopolymer mortars. Exceptionally, the addition of CB indistinctly lowered the densities of the samples. With these findings, it can be said that the adding ratios were chosen appropriately. Ultrasonic pulse velocity (UPV) tests were conducted using ASTM C597 [42]. According to the data from the UPV test, it was understood that the addition of fibers affected the wave flow of the mortars and impacted the matrix’s homogeneity and compactness, as it elevated the UPV of the reinforced samples compared to that in the control one. To determine the abrasion resistance, cube samples of 71 × 71 × 71 mm were cast and tested using BS EN 1338 [43].

With the HD method, if there is a control in the database, the parameters of the alternatives are divided by the parameters of the control. If a control is not present in the database, the mean of the parameters of the other options is first determined. The mean is multiplied by an incrementing constant (i.e., 2, 3, …), and the parameters of the alternatives are divided by the modified mean of the control parameter. Here, it can be recommended that instead of selecting the mean, the value can be chosen as greater than two times the maximum. Then, if weighting of the parameters is intended, the relative parameters are multiplied by the weights of the parameters. In the last part, the scalar value for each alternative is calculated using a fraction numerator and denominator function. The fraction numerator and denominator involve multiplying the relative parameters of the alternatives by less and greater than 1.0, respectively.

At this point, the physical properties, the mechanical properties, the applied current value while electrical curing, and the chemical properties of the geopolymers were used in the HD method to calculate the K values of the geopolymers. In total, 273 parameters were considered. The best geopolymers in each group in terms of the CF, SF, EW, and CB contents and the best of all geopolymers were determined holistically and objectively. The calculation steps for the HD method are given in Appendix A.

## 3. Results

### 3.1. Physical Properties

#### 3.1.1. Bulk Density, Porosity, and Water Absorption

**Figure 3:**
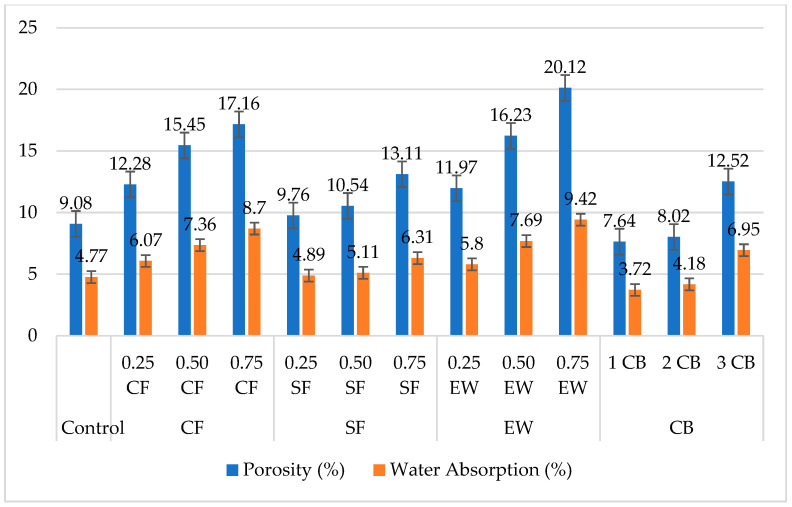
Physical properties of all geopolymer samples.

The results on bulk density, porosity, and water absorption are presented in Figure 3 and Table 4. Adding fibers to the MK-GBFS-based geopolymer mortars significantly impacted the samples’ porosity and water absorption. A relationship was also determined between the water absorption and porosity values. The porosity and water absorption correlation coefficient in this study was calculated as 0.9365, indicating a reasonable degree of correlation and demonstrating the validity of the study results. Adding CF increased the porosity and water absorption compared to those in the control mixture for the three mixes. The addition of SF slightly increased the values, while 0.75% addition of SF increased them a little more. In general, the addition of EW effectively increased the values, while 0.75% addition of EW increased the porosity to 121% and the water absorption to 97% compared to those in the control. For EW addition, it can be said that the physical structure of the fiber easily allows for voids. Meanwhile, 1% and 2% addition of CB slightly decreased the values, while 3% addition of CB increased the results. This fact can be explained by voids thanks to the insoluble structure of CB. Mindess et al. [44] mentioned that the increase in conductivity is due to the presence of water in capillary pores. In line with this information, increasing porosity and water absorption can increase the electrical conductivity and other properties.

To further understand the inherent mechanisms of the described trends, decomposition of the fiber-type and dosage-dependent responses reveals sensitive interactions between the additive characteristics and matrix response. For instance, the rise in porosity of the CF series—from 9.08% in the control to 17.16% at 0.75% CF—suggests not merely volumetric expansion of the pore spaces but an inherent loss of gel continuity, probably owing to negative fiber–matrix interface bonding and irregular dispersion. Microstructural breaks of this kind can provide preferred pathways for moisture, hence the simultaneous rise in water absorption, reaching a maximum at 8.70%—an 82.43% increase. By contrast, the SF series registered only moderate changes, in that the porosity ranged between 9.76% and 13.11%, with a corresponding water absorption up to 6.31%. These relatively restrained responses are characteristic of the better integration of SF into the binder matrix, probably by virtue of improved mechanical hold-down and steadier shearing alignment in the mixer. Advances in the physical properties at 0.75% SF can be explained by virtue of an increased surface area, inducing additional paste demand, if unquantified, that may take the form of increased bleeding or internal air voids.

Interestingly, the EW series produced the greatest aggressive loss in physical work, with the porosity shooting from 11.97% at 0.25% EW up to 20.12% at 0.75%, corresponding to a 121.59% increase versus that in the control. Water absorption correspondingly increased, from 5.80% up to 9.42%, corresponding to a 97.47% increase. These findings suggest the inherent incompatibility of waste erosion wire with the geopolymer gel, almost certainly because of its rough, acicular morphology and surface oxidation, resisting adhesion and mix-redundant air entrapment. The intrinsic rigidity of EW further almost certainly limits the mobility upon compacting, consequently engendering interconnectedness with macroporosity. With the concomitance of high porosity through open void systems, the durability perspective comes to the fore because such systems may catalytically promote the ingression of unwanted agents such as chlorides, sulfates, and CO_2_, lowering the service life unless offset by surface pretreatment or densification schemes. While the CB series presents the bifunctional character of micro-additives as either pore fillers at reduced dosages or agglomerators at increased dosages, the reduction in water absorption and porosity at 1% and 2% CB indicates successful pore filling with enhanced particle packing, likely owing to the ultra-fine dimension and hydrophobicity of the CB particles. The slight increase in porosity and water absorption at 3% CB indicates a dosage-sensitive limit after which the filler’s impact becomes overriding, countered by agglomerative tendencies, creating local weak spots or void clusters. The dosage-sensitive response of this kind thus places significant emphasis on optimizing the content, especially in the framework of inert micro-scale additions into the geopolymer mortars.

Furthermore, the structural transformations registered through water absorption and porosity are not only harbingers of the transport characteristics but are of thermal as well as electrical importance. For applications in systems where functional conductivity (e.g., de-icing, electrical shielding from unwanted radiation/interference (EMI shielding)) holds significance, the increased porosity supplemented by excessive ionic movements through the increase in water absorption can facilitate an electrical response. The same, however, can be the origin of unwanted side reactions in the form of excessive drying shrinkage or efflorescence in open systems.

Ultimately, the similarity of the porosity–water absorption relationship for all of the tested mixtures confirms the efficacy of these gauges as internal matrix structure surrogates. The extremely high correlation coefficient (0.9365) reinforces that internal void structure modifications are faithfully represented by even the simplest of gravimetric water absorption tests, so they are practical surrogates in performance-driven quality control applications. Strong linearity further suggests that changes made to the fiber material or additive complement will systematically impact the two properties in tandem. Thus, strategic mix design must account for not only the mechanical reinforcement specifications but also the inherent impact upon the overall transportation behavior, as well as long-term durability.

#### 3.1.2. Ultrasonic Pulse Velocity

The UPV results are presented in Table 5 at 28 days to evaluate the homogeneity of the geopolymer mortar matrix.

As is well known, the speed of propagation of ultrasonic waves in a cement-based or geopolymeric material is strongly sensitive to the occurrence of microstructural defects such as microcracking, voids, and incompletely bonded zones; smaller UPVs usually correspond to a higher porosity and lower density of the matrix. In the current investigation, such a relationship emerged clearly since most of the fiber-reinforced mixtures had smaller UPVs in comparison with that in the control mixture (3.12 km/s), reflecting the inclination of the incorporation of the fiber to increase trapped air and interfacial transition zone irregularities upon mixing. For instance, CF reinforcement registered a significant reduction in the case of 0.25% CF (2.66 km/s, −14.60%), showing large-scale disturbance of the continuity of the matrix at a low dosage, owing to probable fiber clustering and a bad compacting effect. Notably, the optimum effect emerged for 0.50% CF, where the UPV increased to 3.32 km/s (+6.50% for control), showing a better fiber distribution and probable densification of the matrix according to the better stress distribution in the early setting; however, for 0.75% CF, the UPV decreased again to 2.91 km/s (−6.84%), probable owing to excessive fiber material causing an increase in void formation. On the contrary, the incorporation of steel fiber (SF) additives consistently improved the UPV over that in the control, where 0.25% SF registered 3.21 km/s (+2.98%), with 0.50% SF showing the highest overall UPV of 3.59 km/s (+15.11%), and with 0.75% SF still showing a comparative value of 3.34 km/s (+6.95%) as a high UPV. Such improvement agrees with the larger stiffness of the SF, which may lead to improved transmission of the stress waves under the condition of a good bond of the fiber–matrix kind and depending on the geometry of the fiber, which can facilitate improved packing without the excessive formation of voids. Waste erosion wire (EW) fibers registered a transition pattern of behavior where 0.25% EW appeared slightly low compared to the control value (3.03 km/s, −2.97%), but considerable improvements for 0.50% EW (3.47 km/s, +11.24%) and 0.75% EW (3.54 km/s, +13.33%) were seen, indicating that adequate dosing of the EW enhances the interlocking of the matrix and reduces the volume of the voids. In contrast, CB modification had mixed results: although the fine particle size of CB could, in theory, occupy microvoids, the UPV in 1% CB was marginally low compared to that in the control (3.08 km/s, −1.38%), and subsequent reductions were noted in the 2% CB (2.95 km/s, −5.56%) and 3% CB (2.97 km/s, −4.77%) treatments. This decrease at the higher CB concentrations most likely results from particle agglomeration, which can form micro-defect clusters and diminish effective transmission routes for the ultrasonic waves. Overall, the results suggest that although some fiber types and dosages (especially optimal levels of SF and CF or EW) have the potential to improve the UPV by optimizing the matrix densification and interfacial adhesion, excessive reinforcement or filler levels may have adverse effects on homogeneity and are in line with previous research showing that fibers are primarily responsible for controlling ultrasonic wave travel in geopolymeric composite material [45].

#### 3.1.3. Abrasion Resistance

**Figure 4:**
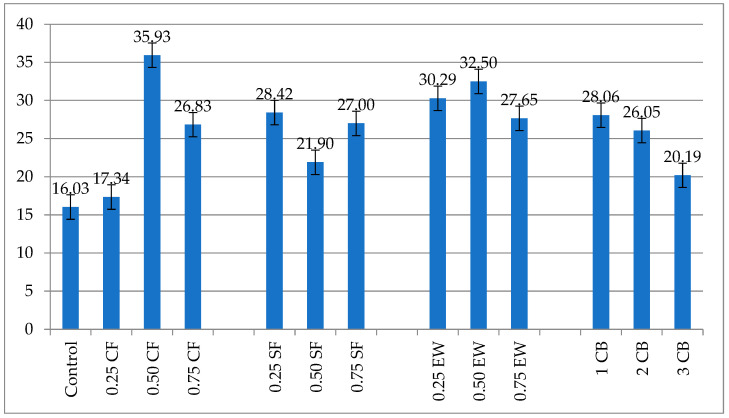
Abrasion resistance results (units are in cm^3^/50 cm^2^).

Figure 4 presents the 28-day abrasion resistance of the geopolymer mortars treated with three various kinds of fiber reinforcements, namely CF, SF, and EW, as well as with CB at three different dosages (1%, 2%, 3%). Generally, all of the treated specimens exhibited an improved performance compared with that of the unreinforced control mix (16.03), therefore confirming the vast potential of these materials to enhance surface resistance for an abrasive surface. The extent of the improvement, however, significantly hinged upon the additive materials’ physical and electrical attributes and their compatibilization within the geopolymeric gel, as well as their respective dosages.

Of all the samples, the highest-performing was 0.50% CF, achieving the highest abrasion resistance value of 35.93, representing a 124% rise in relation to that for the control. It was closely followed by 0.50% EW at 32.50 (103% increase), thus placing the two in the highest-performing category. These two mixes systematically surpassed all of the others in their absolute values as well as relative effectiveness, exhibiting the best combination of mechanical reinforcement, dispersion, and electro-conducting curing compatibility. CF’s length-to-diameter ratio, in conjunction with its higher tensile strength and conductivity, likely allowed for effective crack-bridging, as well as promoting denser growth of the microstructure, especially in the early curing stage. Accordingly, EW, being less refined in its morphology, allowed for good mechanical anchoring by virtue of its irregular, coarse texture, supplementing the abrasion resistance according to the ability to initiate mechanical interlocking, as well as resisting detachment of the particles.

A comparative list of all of the peak-performance samples (by additive and optimum dosage) follows:

0.50% CF (35.93) > 0.50% EW (32.50) > 0.25% EW (30.29) > 0.25% SF (28.42) ≈ 1% CB (28.06) > 0.75% SF (27.00) > 0.75% EW (27.65) > 0.75% CF (26.83) > 2% CB (26.05) > 0.50% SF (21.90) > 3% CB (20.19) > 0.25% CF (17.34) > Control (16.03).

On this basis, three different levels of performance can be differentiated:

Higher abrasion resistance (best quality): 0.50% CF;

The middle tier (moderate gains): 0.25% EW, 0.25% SF, 1% CB, 0.75% EW, 0.75% SF;

The low-performance or overdosage-affected tier: 0.75% CF, 2% CB, 0.50% SF, 3% CB, 0.25% CF.

CB’s example, in turn, is extremely representative. While 1% CB kept up the performance of the SF at 0.25%, higher dosages reduced its effectiveness up to a 20.19 value at 3%, only 26% better than the control. This shows the presence of a saturation threshold after which CB agglomerates and induces disruption of the matrix continuity rather than improving it. It follows the trend of excessive use of the nanoparticles, in which the beneficial characteristics of pore filling and densification are offset by the incompatibility of the interface, with the creation of weak zones.

Statistical analysis was conducted utilizing one-way analysis of variance (ANOVA) to investigate the effects of fiber type and dosage on the surface electrical resistivity and compressive strength, and then, Tukey’s Honestly Significant Difference (HSD) test was conducted for the post hoc test (α = 0.05, n = 3) to identify differences between pairs of groups. ANOVA demonstrated that the twelve groups differed from one another significantly (F(11, 24) = 11.48, *p* < 0.001) in their mechanical and electric behavior in the geopolymer mortars. Importantly, the variations among the groups indicated by the ANOVA were analyzed with the use of Tukey’s HSD multiple comparisons, which displayed that the compressive strengths of the 0.50% EW and 0.50% CF combinations significantly improved in comparison with those for the control and most of the low- and medium-fiber-dosage groups and therefore indicated their optimum contribution to matrix compaction and load conveyance. Conversely, the 3% CB mixture was in the lowest statistics group at all times, indicating the negative contribution of highly conductive fillers to the mechanical performance. Also, the outcomes strongly showed that fibrous or conductive fillers at an intermediate level of dosage (0.50%) provided the highest statistically significant increments in surface resistance, affirming the synergistic contribution of intermediate levels of fibers to improving both the structure and electromechanical response in geopolymers. The levels of significance in the groups were also determined from Tukey’s test and critical values from 95% intervals of confidence, allowing room for intensive numerical analysis for differentiation of the blend performance and verification of the observed experimental trends. According to Ribeiro et al. [7] and Farhan et al. [11], the addition of fibers enhances the mechanical strength by virtue of stress redistribution and crack-bridging [7,11]. Besides that, Payakaniti et al. [6] also indicated that electrically conductive additives such as CF and EW enhance the strength of the matrix, in addition to enhancing the efficacy of electrocuring, through promoting localized heat evolution as well as ion mobility in the process of geopolymerization. The twin functionality of mechanics–electrics offers the rationale behind the exceptional workability of 0.50% CF as well as 0.50% EW in the present work. Interestingly, the optimum dependency in the synergy of conductivity, aspect ratio, and optimum dispersion seems to be the overriding determining factor in the optimization of abrasion resistance.

### 3.2. Compressive Strength Results for Samples with Different Molarities Within the Scope of Preliminary Tests

**Figure 5:**
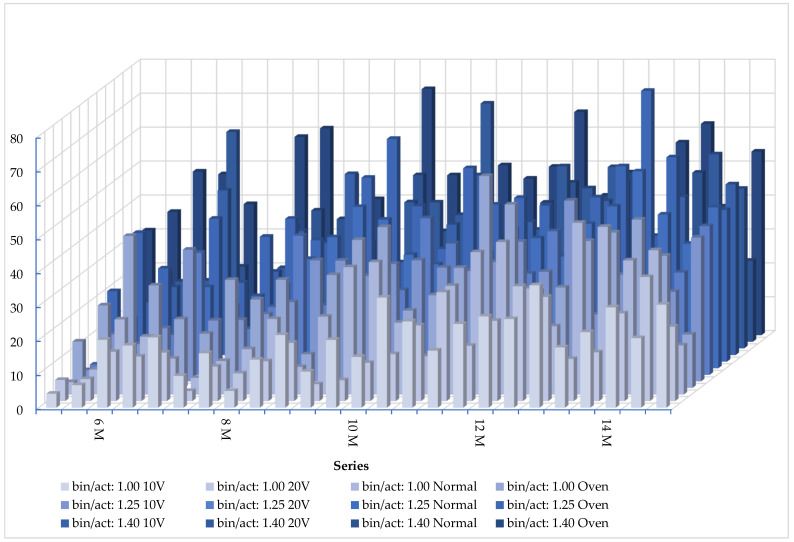
Compressive strength results of preliminary tests.

Figure 5 demonstrates the compressive strength results for the geopolymer samples at five different molarities of SS for three various B/A ratios. It can be said that the highest value for electrical curing was obtained from the 0MK100S sample, which was 20.68 MPa at 10 V, while the values for the other samples were slight and similar. Moreover, the 10 V electrical curing value for 0MK100S was higher than that under ambient and oven curing. This evidence displays that while the molarity of SS was low, electrical curing was suitable. It could be seen that the highest values for all curing methods were obtained using 0MK100S at the 1.25 B/A ratio. The highest value for electrical curing was for 0MK100S at 38.80 MPa at 10 V, while the values for the other samples were slight and similar. This value simply indicates that electrical curing at a low voltage can be beneficial with a lower molarity and amount of activator. It could be seen that the highest value for electrical curing was achieved for 0MK100S at 63.80 MPa and 20 V for the 1.40 B/A ratio. At the same time, both results under electrical curing were also higher than those under ambient and oven curing for 0MK100S. At the same mixing proportion, a rise in the B/A ratio was very effective as reflected in the electrical curing results. The gain in strength for the 100% MK samples was weak under the electric and ambient curing methods. It has been stated that using MK alone makes for a weak structure without heat treatment to high degrees by Yun-Ming et al. [14]. On the contrary, the strength of the 100% GBFS samples was remarkable, although the molarity of NaOH was very low.

The highest values for electrical curing were 21.38 MPa at 10 V and 0MK100S at 8 M, while both results under electrical curing were also higher than those under ambient and oven curing for 0MK100S. It can be ascertained that the results were increased by the amount of GBFS. The highest value for oven curing was obtained using 50MK50S for the 1.25 B/A ratio. The highest value for electrical curing was obtained at 40.95 MPa using 25MK75S at 20 V, and this result was higher than those under ambient and oven curing. It could be seen that the highest value for electrical curing was obtained from 0MK100S at 20 V. Moreover, the value for electrical curing of 0MK100S was 53.34 MPa at 10 V, which was higher than that under ambient and oven curing. In general, the results of electrical curing were close to those of ambient and oven curing. The results at 8 M were mainly similar to those at 6 M for all curing methods. The highest value for electrical curing was obtained for 25MK75S at 32.45 MPa and 10 V and 10 M for the 1.00 B/A ratio. This result was higher than those under both ambient and oven curing methods. Moreover, both values under electrical curing for 0MK100S were higher than those under ambient curing. It can be seen that the highest value for electrical curing was obtained using 25MK75S at 20 V and 46.12 MPa for the 1.25 B/A ratio. This result was higher than those achieved under ambient and oven curing. At the same time, the values under electrical curing for 50MK50S at 10 V and 0MK100S at 20 V were higher than those under ambient curing. It can be seen that the highest value for electrical curing was obtained with 0MK100S at 72.18 MPa and 20 V for the 1.40 B/A ratio, and this value was the highest result of all electrical curing tests. This result was much higher than those under ambient and oven curing. It was observed that all values for electrical curing at the two voltage rates were similar to those under ambient and oven curing.

It can be observed that the electrical curing values of 0MK100S were 36.06 MPa for 10 V and 30.69 MPa for 20 V at 12 M for the 1.00 B/A ratio. These results were higher than those under ambient and oven curing. Meanwhile, the value of 25MK75S at 20 V, 33.84 MPa, was also higher than that under the other curing methods. It can be seen that the highest value with the three B/A ratios was obtained as 53.28 MPa for 0MK100S at 10 V for the 1.25 B/A ratio. It was observed that these values for electrical curing were slight and similar to those at other ratios and higher than those under ambient and oven curing in all samples. It can be seen that the highest values for the geopolymer mortars under all curing methods were obtained with 50MK50S at the 1.40 B/A ratio. The highest value for electrical curing was obtained as 53.68 MPa at 20 V. It was observed that the value for electrical curing was similar to that with the oven curing method and higher than that under ambient curing. It is easily understood that as the ratio of binder to activator increases, the mechanical strength of electrical curing becomes stronger.

Equally, the highest value for electrical curing was 36.56 MPa at 20 V for 25MK75S at 14 M, while the ambient and oven curing results were similar for the 1.00 B/A ratio. Both results for electrical curing were also higher than those under ambient and oven curing for 0MK100S. It can be seen that the highest values for electrical curing were 43.80 MPa at 20 V for 0MK100S, while both results of electrical curing were also not far from those under ambient and oven curing for 0MK100S at the 1.25 B/A ratio. It can be seen that the highest values of electrical curing were 57.20 MPa at 20 V for 25MK75S at the 1.40 B/A ratio, and this value was much higher than those under ambient and oven curing. Meanwhile, the electrical curing results were much higher than those under ambient curing for 0MK100S. As Farhan et al. [11] have previously mentioned, when the molarity of NaOH rises from 6 M to 12 M, the strength of geopolymer mortar rises. This research determined that mortars made with 6 M and 8 M had low mechanical properties. It was believed that the low-alkali source may have been the reason for this fact. Conversely, the mortars with 10 M, 12 M, and 14 M had much better mechanical properties for all curing methods. Low molarity gives insufficient Na^+^ ions to complete polymerization, and higher molarity than the optimal level results in undesirable polymerization because of its high viscosity. Increasing the B/A ratio at a constant molarity caused a higher strength but lower workability. Mainly, producing electrically cured 100% MK samples was hard because of agglomeration. Fast heating using electricity gave rise to drying of the samples and cracks. This was because clay has a layer-like structure that reduces the workability of the mortar. Heah et al. [13] stated that MK has a higher water demand because of this fact. Davidovits [46] agrees that MK cannot be compacted, but it maintains its structure after geopolymerization, and the reaction occurs at the surface of the geopolymer. Clay minerals have rich Al_2_O_3_ and SiO_2_ contents, with the total composition of both in the 70–90% range, which varies depending on the location’s origin and geology [4,13]. MK usage requires a high-alkali medium [14,47]. For the 100% GBFS samples, increasing the bin/act ratio achieved a high strength. Aziz et al. [40] studied that the ideal ratio was 3.0 for slag. The most advantageous trials were for seen 50MK50S at 20 V, 1.40 bin/act, and 12 M in terms of strength, economy, and ecology. Tuyan et al. [48] explained that reducing the water-to-binder ratio increases the strength. Dan et al. [4] studied the early strength of MK-GBFS-based geopolymers and suggested that a GBFS content of 60% was an ideal mix [4]. In this way, MK and GBFS performed well at a low voltage and molarity. This mixing ratio was applied in the subsequent phase of the study.

### 3.3. Mechanical Properties of Fiber-Added Samples

#### 3.3.1. Compressive Strength

**Figure 6:**
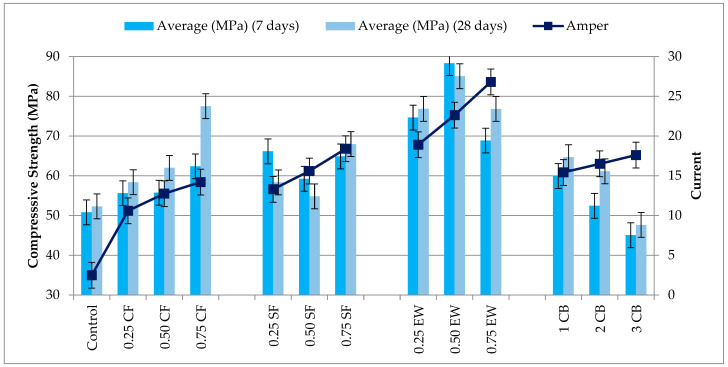
Compressive strength and maximum current values.

Figure 6 provides a high-resolution illustration of the relationship between the evolution in the compressive strength and peak current of electrocured geopolymer mortars, presenting an exceptionally rare quantitative coincidence of mechanical and electrothermal performance metrics. Fiber-reinforced series of all strengths substantially surpassed the control, which measured a respectable 50.82 MPa at 7 days and 52.29 MPa at 28 days, corresponding to the lowest peak current of 2.50 A. Even the poorest-performing fiber system at a reduced dosage provided at least a 10–25% strength improvement compared to the control at 7 days, further confirming the embedded advantage of fiber-mediated electrical conduction in compacting the matrix. In the CF series, the incremental addition of fibers from 0.25% to 0.75% increased the 7-day strength from 55.63 MPa (+9.5%) to 66.14 MPa (+30.2%) and the corresponding current from 10.60 A (+324%) to 14.20 A (+468%). Most remarkably, the 0.75% CF mix defied the characteristic geopolymer plateau effect by enhancing the 28-day strength to a remarkable 77.49 MPa, an impressive 48.1% improvement compared to the control, repeating Dogan et al.’s [49] finding of the maintained conduction and crack-bridging capability of dispersed CF networks beyond the initial curing period. For SF, the 7-day strength rose from 62.00 MPa (+22.1%) at 0.25% to 74.62 MPa (+46.8%) at 0.75%, with corresponding peak currents of 13.30 A (+432%) and 18.40 A (+636%), respectively. Although small drops in 28-day strength were recorded in the lower-dosing case (e.g., −2.6% at 0.25 SF), the net gain was generally substantial, consistent with Dehghanpour et al. [50], who noted that steel fibers have the capability to reduce the resistivity of concrete by an order of magnitude or more, corresponding to improved thermal uniformity and bond development.

CB exhibited a non-linear response: 1% CB raised the 7-day strength to 59.93 MPa (+17.9%) with an elevated current of 15.42 A (+516%), but raised percentages (2% and 3%) showed reductions to 52.40 MPa and 45.05 MPa, representing only +2.98% and −11.4% changes compared with the control, respectively. The loss of strength is attributed to similar patterns to those for particle agglomeration and differential heat distribution, potentially causing localized temperature gradients detrimental to gel continuity. EW was the undisputed champion. At 0.25% EW, the 7-day strength was 76.82 MPa (+51.2%) and 28-day 85.06 MPa (+62.6%), with an 18.90 A (+656%) max current. The 0.50% EW form established the dataset’s peak, 88.30 MPa at 7 days and 85.06 MPa at 28 days—with +73.82% and +62.6%, respectively, increases compared to the control—while still carrying a 26.80 A (+972%) current. Even at 0.75% EW, where slight setbacks in strength to 76.80 MPa (28 days) were recorded, the gain was still +46.87%, with the strength. The coincidence of the highest current (26.80 A) and one of the highest strength results (0.50% EW) further supports the proposal of an optimally percolated conductive network maximizing both the thermal and mechanical results. As noted by Payakaniti et al. [6], in the case of CF-reinforced materials, the two-fold function of conductive fibers of boosting the reaction kinetics and securing microstructural integrity under electrical curing provides a positive feedback loop—increasing current aids in better geopolymerization, aiding in greater conductivity due to reduced porosity and microcracking.

#### 3.3.2. Flexural Strength

**Figure 7:**
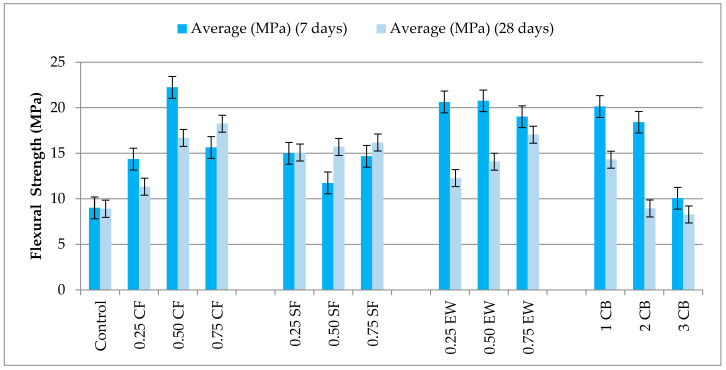
Flexural strength results.

Figure 7 reports the development of the flexural strength of the 7- and 28-day-aged geopolymer mortars subjected to electrical curing, pointing out that the adoption of conductive and semi-conductive fibers profoundly alters the bending response concerning the unreinforced control mixture, which only reached 9.00 MPa at 7 days and 8.90 MPa at 28 days, characteristic of the brittle fracture mode common in geopolymer matrices without crack-bridging components [26]. The overall behavior among all types of fibers reveals that the early-age bend strength has a close correlation with fiber conductivity, such that the best 7-day results were consistently represented by the most conductive types of fibers. This correlation emerges even more noticeably if some of the highest 7-day results are compared, such as 0.50% CF (22.24 MPa, +147.1%) and 0.50% EW (20.76 MPa, +131.8%) and their corresponding lower-conductivity analogues in the SF series, which only reached 15.00–16.17 MPa (+66.7–79.7%) in the same duration. Nonetheless, the retention behavior after 28 days shows a more nuanced correlation which cannot be addressed by conductivity alone. In the CF series, although the early-age strengths were among the highest (0.25% CF: 14.36 MPa, +59.6%, 0.75% CF: 18.25 MPa, +103.0%), the retention ratios were only 75–86%, with 0.50% CF decreasing to 16.69 MPa (−24.9%) and 0.75% CF to 15.64 MPa (−14.3%). This drop implies CF’s ability to hasten early gel development due to localized concentration of heat, but because of CF’s smooth surface topography and limited mechanical anchorage, interfacial debonding and microcrack initiation are allowed under prolonged stress cycles. Conversely, SF, even with lower electrical conductivity, showed better long-term stability; 0.25% SF improved slightly from 15.00 MPa to 15.08 MPa (+0.53%), and 0.50% SF and 0.75% SF only experienced slight reductions (−6.6%, and −9.3%, respectively). This characteristic indicates SF’s hooked-end topography and mechanical interlocking as important mechanisms, where even if the matrix experiences microstructural relaxation once past the peak hydration–geopolymerization period, SF still ensures effective bridging of cracks, and the energy dissipation capacity is maintained.

These EW series are characterized by dual behavior: 0.25% EW showed an excellent initial strength of 20.63 MPa (+129.2%) but showed a remarked deterioration to 12.28 MPa (−40.5%), indicating that even at lower volume fractions, though early acceleration by conductivity is sufficient, poor fiber density hinders late-life crack-bridging efficiency. Conversely, 0.50% EW showed an excellent initial strength (20.76 MPa) and good retention at 28 days (19.01 MPa, −8.4%), achieving the best combination of conductive network integrity, mechanical reinforcement, and quality of dispersion. However, 0.75% degraded more markedly (−17.4%), and at system levels greater than the optimum, higher volumes of fibers may induce clusterization of a localized nature, limiting even stress transfer uniformity and promoting premature matrix microcracking.

The CB-reinforced mortars exhibit the most non-linear dosage response. The 1% CB delivered 20.13 MPa (+123.7%) at 7 days and retained 18.41 MPa (−8.5%) at 28 days, indicating that a low CB content can form efficient conductive pathways without severely disrupting matrix continuity. However, higher CB dosages produced a sharp deterioration: 2% CB declined from 14.29 MPa (+58.8%) to 8.94 MPa (−37.4%), and 3% CB declined from 10.05 MPa (+11.7%) to 8.27 MPa (−17.7%). This drop aligns with the known agglomeration tendency of fine carbon particles, which creates weak interfacial zones and uneven resistive heating, leading to heterogenous microstructural densification and a reduced flexural load-bearing efficiency.

Cross-comparison with compressive strength data reveals critical divergence: although the top early flexural strengths coincide with some of the strongest compressive strengths, generally, the flexural strength has greater retention ratios 28d/7d (e.g., SF 0.25%: 1.005; EW 0.50%: 0.916) compared to those for compression. This means that even when matrix densification has plateaued or slightly retreated, the reinforcement by fibers still maintains load-carrying after a crack through mechanical bridging. The dataset thus defines a two-parameter optimization principle: a good early-age flexural capability tends to be controlled solely by the conductivity of the fibers and the continuity of their network, but long-term retention relies on mechanical anchorage, the quality of dispersion, and the integrity of the fiber–matrix bond interface.

From this, 0.50% EW comes out as the best balanced reinforcement, achieving fast stiffness accrual and a durable bending strength; SF provides superior long-term stability in a lower-conductivity system; CF has the best early-age acceleration but needs better interfacial treatments to retain it; and CB must be strictly controlled in dosage lest the system performance collapses.

#### 3.3.3. Splitting and Direct Tensile Strengths

**Figure 8:**
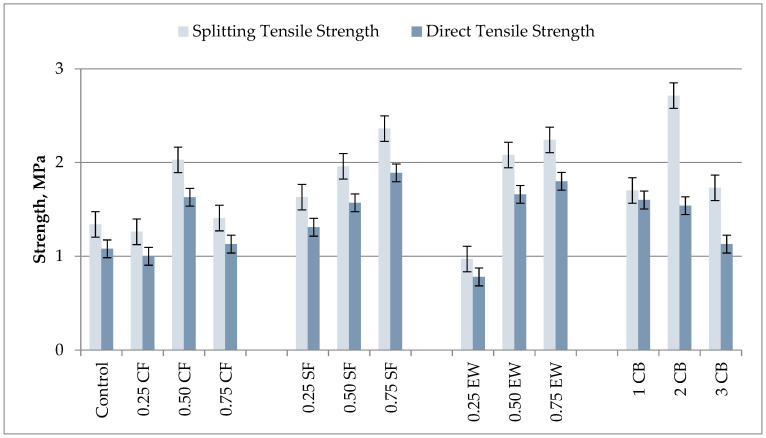
Splitting tensile and direct tensile results.

Figure 8 presents the 28-day results for the geopolymer mortars’ splitting tensile strength (STS) and direct tensile strength (DTS), giving an exhaustive comparison of the effect of fiber type and dosage effect on tensile performance. Practically all of the fiber-added samples showed elevated and broadly parallel enhancements in both tensile strength properties with respect to those of the control (STS = 1.34 MPa, DTS = 1.08 MPa), suggesting that fiber reinforcement effectively boosted the crack-bridging capability and slowed down crack-propagation, leading to fracture. The only exception was the 0.25% EW-added sample, which measured significantly low tensile properties (STS = 0.97 MPa, DTS = 0.78 MPa), corresponding to reductions of about −27.6% and −27.8% compared to those of the control, respectively. This downturn may be attributed to an insufficient amount of fibers to establish an interconnect reinforcing network, besides likely dispersion problems giving rise to a localized rise in stress. Even so, an increase in EW addition resulted in considerable enhancements, with 0.50% EW attaining 2.08 MPa (+55.2%) in STS and 1.66 MPa (+53.7%) in DTS and 0.75% EW attaining 2.24 MPa (+67.2%) in STS and 1.80 MPa (+66.7%) in DTS, unequivocally surpassing the control and demonstrating a threshold level of dosing whereby the fibers can actively participate in tensile load transmission. The highest individual performance was achieved with a 2% CB sample, which achieved an STS of 2.71 MPa (+102% above control), the highest value in the dataset, whereas its DTS was 1.60 MPa (+48.1%). The connection between STS and DTS mainly results from intrinsic variations in the distribution of the stress, the mechanics of fracture, and the microstructural interaction mechanisms involved in each test setup. A wide-range compressive load distribution over a prismatic or cylindrical test piece under an STS test produces indirect biaxial tensile stress conditions along the central axis, giving a non-uniform but highly distributed triaxial stress condition. This distributed stress condition reduces the local stress concentration and induces the gradual growth of a number of microcracks across the test piece, leading to sequential fiber enthalpies and distributed bridging of the cracks. Fine conductive fillers, i.e., carbon black, also densify the matrix and improve fiber–matrix contacts to toughen the interfacial transition zones (ITZs) for gradual contacting. Accordingly, STS testing typically registers higher apparent ductile values because the composite resists possible failure due to the bulk contribution of microcrack interaction, confinement, and fiber bridging. DTS testing, however, imposes direct uniaxial and concentrated uniaxial tensile stress along the longitudinal axis of the test piece; this causes a dominant single fracture plane that constrains the extent of fiber incorporation and the dissipation of stored energy. Upon nucleation and propagation of the major crack, the lack of a laterally confined load results in a rapid loss of load-bearing capacity and sudden breakdown. Additionally, another variable that impacts the results further is that of specimen geometry and size [23,29] because specimens with a higher DTS have naturally higher micropores and lower fiber alignment and hence higher chances of early crack initiation. STS specimens have a more widespread stress profile that provides them with higher ductile values. Under the conditions of the electrothermal cure test, this disparity is further aggravated because the current density and the consistency of the temperature are reduced by the increase in the specimens’ lengths, such that less consistent gel maturation in the DTS specimens occurs. On the contrary, the systematic variation between STS and DTS in this test is a consistent result of the varied stress paths according to the fracture geometries and microstructural interactions, which are unique for each test condition: lower-STS samples registered a greater uniform thermal field that supplied a denser gel growth for higher cohesivity between the fiber–geopolymer matrix such that the STS/DTS ratios fell between the values of 1.2 and 1.4, which are standard responses of fiber-reinforced geopolymer mortars.

#### 3.3.4. Microstructural Analysis

FTIR, XRD, SEM, and EDS/EDX analyses were performed together on 28-day samples to elucidate the microstructure of the geopolymer mortars. The specimens for these analyses were extracted from the cracked samples after the compressive strength tests.

##### Fourier Transform Infrared Spectroscopy

**Figure 9:**
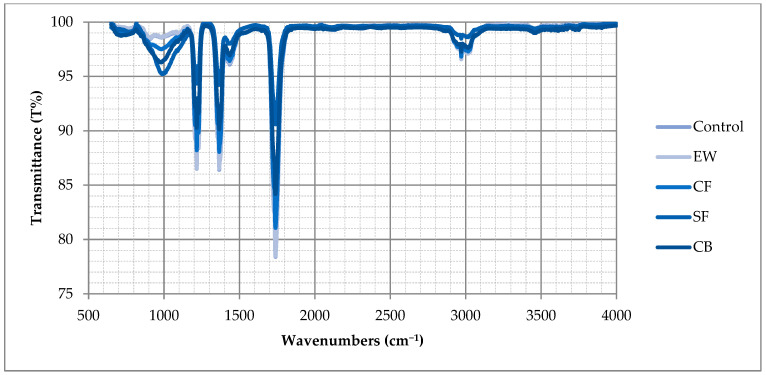
FTIR spectra of MK-GBFS geopolymer samples.

Mineralogy is also significant for the synthesis of geopolymer mortars, and Figure 9 shows the FT-IR spectra of the synthesized samples, scanned between 4000 and 650 cm^−1^ at a spectral resolution of 4 cm^−1^ using a PerkinElmer Spectrum 100 (PerkinElmer Inc., USA) FT-IR spectrophotometer. According to the analysis, adding selected fibers had no effect, and using different fibers did not make a significant difference in the polymerization process [25]. According to the literature, clay-based geopolymers show leading narrow bands around 990 cm^−1^ and 1200 cm^−1^, with asymmetric stretching vibrations of Si-O and Al-O bonds. The band in the 1300–1400 cm^−1^ range was characteristic of C-O bonds. The 3500–4000 cm^−1^ peaks and about 1700 cm^−1^ were associated with stretching O-H bonds [12]. The results followed those of previous studies [51,52,53].

##### X-Ray Diffraction

An XRD analysis of the chemical composition of both the control and reinforced geopolymer mortars was conducted. The readings were obtained using a Malvern PANalytical Empyrean MultiCore diffractometer (Malvern Panalytical Ltd., UK) with a Cu Kα radiation source (λ = 1.54059 Å). The machine itself was set at a constant operation of 40 kV and 40 mA, which are standard laboratory parameters for a source of Cu Kα radiation, and readings were acquired for a 2θ value ranging between 2° and 120° at a scan speed of 1°/min. The test samples were powdered before being sent for testing.

**Figure 10:**
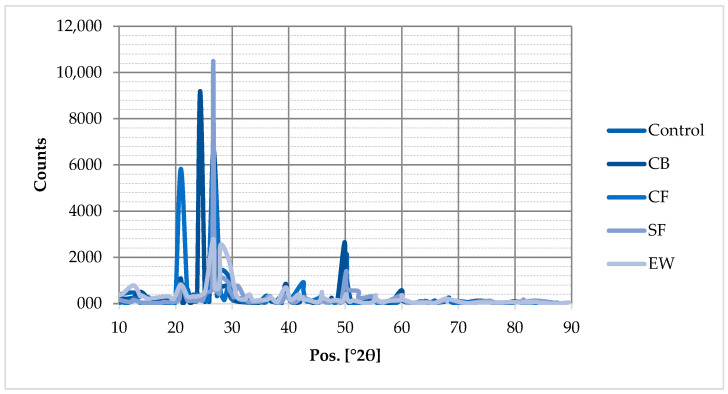
X-ray diffraction patterns of MK-GBFS geopolymer samples.

The XRD patterns of all samples are depicted in Figure 10. When the XRD data were analyzed, quartz peaks were found in all geopolymer mortar samples. The quartz peaks had a higher intensity in the ~22–35° of 2θ range. As stated in the literature, these quartz peaks are highly caused by the binding materials’ high crystalline silica content [10,26,53]. The 2θ peaks in the XRD patterns of the MK-GBFS-based geopolymer mortars between ~22 and 35° indicate effective geopolymerization [26,54]. Consequently, the chemical composition of the MK-GBFS-reinforced geopolymer mortars was unaffected by the fibers’ presence. The other peaks showed unreacted geopolymerization for all samples [34].

##### Scanning Electron Microscope

**Figure 11:**
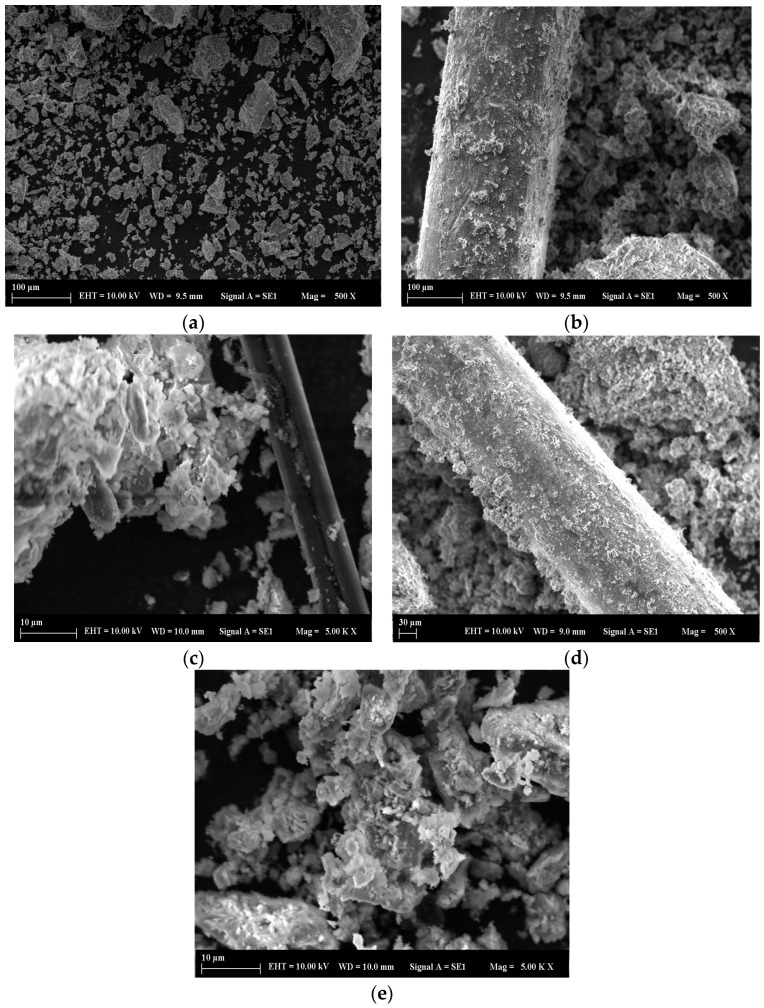
Scanning Electron Microscope micrographs: (**a**) control, (**b**) EW, (**c**) CF, (**d**) SF, and (**e**) CB.

Scanning Electron Microscope (SEM) analyses were performed to analyze the microstructure of the manufactured reinforcement geopolymer mortars and to explore the bonding between the matrix and fibers. Before imaging, small amounts were removed from the broken surfaces of the 28-day specimens after compressive strength testing, dried out in an oven for 24 h, mounted onto aluminum stubs using conductive carbon tape, and sputter-coated with a thin (~10 nm) gold coat to avoid charging and enable clean imaging. The surface microstructure of the samples was investigated using a Zeiss EVO LS 10 model. The analysis was conducted at a 10.00 kV acceleration voltage with a 9–10 mm working distance. Micrographs at various magnifications for the control and SF-, CF-, CB-, and EW-added samples are shown in Figure 11 at the age of 28 days. Consequently, it was observed that all samples exhibited a dense microstructure, which showed fertile geopolymerization of MK and GBFS. The SEM images revealed a clear and properly organized geopolymeric matrix that indicated successful microstructural development and sound curing, and the results were in agreement with the mechanical properties, XRD, and FTIR tests, producing a consistent picture of the reaction procedure.

##### Energy-Dispersive X-Ray Spectroscopy

**Figure 12:**
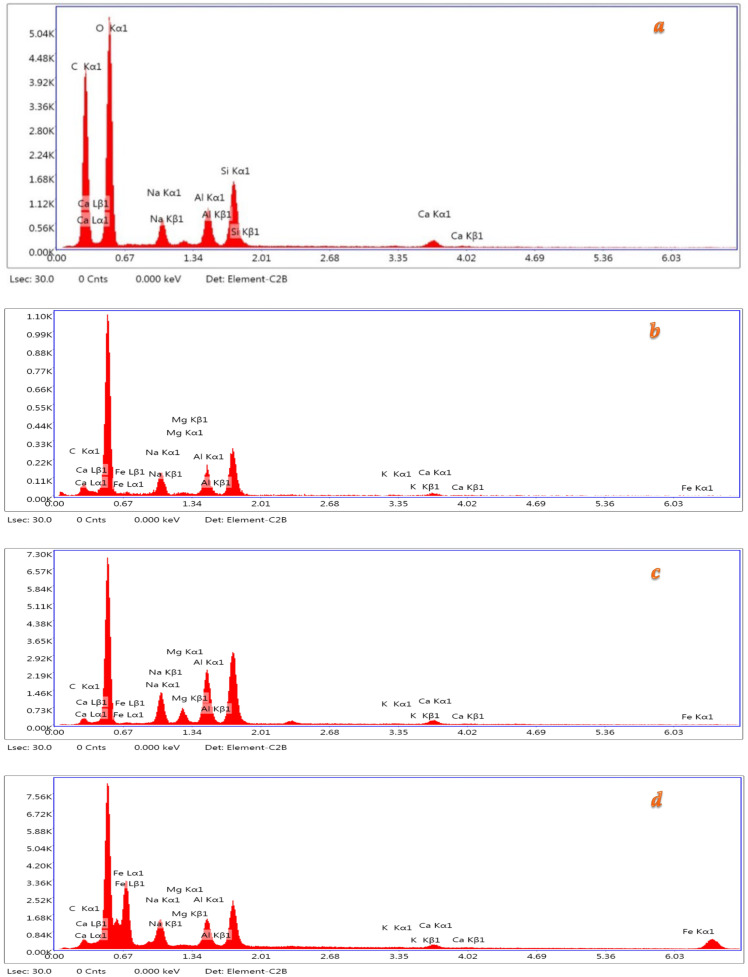

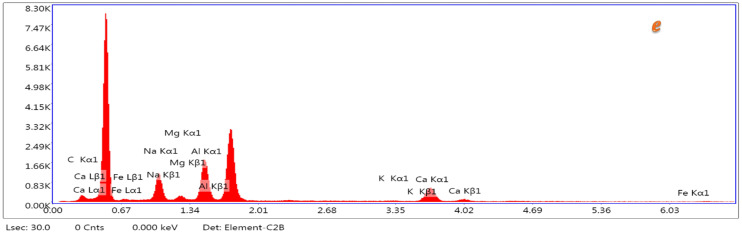
Energy-Dispersive X-Ray Spectroscopy: (**a**) control, (**b**) EW, (**c**) CF, (**d**) SF, and (**e**) CB.

EDS line scanning was carried out for the control, fibrous (SF, CF, EW), and particulate microfiller (CB) reinforcements. The analysis was conducted using a Thermo Scientifıc Apreo 2S LoVac (Thermo Fisher Scientific Inc., USA) model at a 10.00 kV acceleration voltage with a magnitude of 1000. Table 6 and Figure 12 represent the EDS results of the control and reinforced samples containing different fiber and microfiller types. The EDS spectrum shows both the chemical composition of the samples and differences between them, like other microstructural tests. It was observed that N-A-S-H gels were formed with C-A-S-H gels in the presence of GBFS. Al was determined as one of the main elements, following C and Na. It has been declared that adding Ca lowers the resistance of N-A-S-H to the liquid environment and prevents it from transforming into a gel layer with a dense structure. Therefore, the gel structure is constantly degraded in a fluid environment [50]. Metal elements were commonly detected on the fiber-added samples. The SEM and EDS analyses were consistent with the mechanical results.

### 3.4. Evaluation of Test Results with the Multi-Criteria Decision Support Method (HD Method) and the Selection of the Best Geopolymer Mortars

Holistic evaluation is an evaluation approach in the literature, and decision support methods ease the evaluation of a database of experimental results. The normally and commonly preferred approach depends on a single-parameter evaluation or a dual-parameter evaluation. In the single evaluation approach, the results are evaluated according to the control and the results from similar papers. This approach was followed in this paper, as mentioned in the previous sections/sub-sections. In addition, a correlation analysis is usually used to find/investigate the linear or non-linear relationship between the parameters in the dual-parameter evaluation approach. In the holistic evaluation approach, independent of the number of parameters, all parameters are used in the calculation steps, and as a result, a constant is usually found. In this way, the constant is used to find the best solution. The HD method is one of the multi-criteria decision support methods used to evaluate alternatives and is used for all parameters. Within the HD method, all parameters of measurements and physical and mechanical properties were given equal weights. This was a decision made to avoid subjective biases due to the arbitrary give-and-take of some parameters over others. Since the experimental program was designed to present equivalent and balanced datasets for each property, employing similar weights made the final value of K* reflect the actual overall performance of each mixture. That is, each parameter played an equal part in the overall decision function, and the mixtures were ordered based on their collective behavior for all properties measured. According to the HD method results, the 0.50 CF, 0.75 SF, 075 EW, and 1 CB specimens were obtained as the best series regarding the CF, SF, EW, and CB contents. On the other hand, the best of all geopolymers was determined to be 0.75 EW. The comparison of the properties of the control and the 0.75 EW geopolymers is given in Table 7. Table 8 indicates that 0.75 EW was superior in terms of strength in 8 of 11 properties Although comparatively less porosity and abrasion resistance were seen for 0.75% EW, its superior mechanical and electrical performance resulted in its selection as having the highest overall composition based on the equally weighted HD method, although its comparatively lower porosity-related performance is noted as a potential improvement area for future studies.

### 3.5. Environmental Impact Assessment of Different Curing Regimes and Reinforcement Types

A life cycle assessment (LCA) was carried out to evaluate the environmental performance of MK–GBFS-based geopolymer mortars incorporating different conductive fibers (CF, SF, EW, and CB) and cured through three regimes—electrical curing at 10 V and 20 V and conventional oven curing at 70 °C—focusing on their energy demand, CO_2_ emissions, water use, soil pollution, eutrophication potential, ozone depletion potential, and toxicity (Table 9). The assessment, normalized to a functional unit of 1 m^3^ of hardened mortar, revealed strong interdependence between the curing method, energy consumption, and cascading impacts across all environmental categories, indicating that curing is the dominant life cycle hotspot. Across all series, oven curing systematically exhibited the highest absolute energy demand of 8000 kWh/m^3^, generating 4000 kg CO_2_/m^3^ alongside 560,000 L of water consumption, 9600 kg of soil pollutant equivalents, 1600 kg PO_4_ eq of eutrophication potential, 0.16 kg CFC-11 eq of ozone depletion, and 120 kg DCB eq of toxicity, serving as the baseline conventional scenario. In contrast, electrical curing at 10 V consistently reduced the energy use by 40–85% depending on fiber type, while 20 V curing approximately doubled the 10 V figures due to higher current intensities. For example, the control mix consumed 1440 kWh/m^3^ at 10 V and 2880 kWh/m^3^ at 20 V, while oven curing required 8000 kWh/m^3^; similarly, 0.50 CF consumed 7164 kWh/m^3^ at 10 V and 14,328 kWh/m^3^ at 20 V, whereas 0.50 EW showed the highest electrical energy use—12,864 kWh/m^3^ at 10 V and 25,728 kWh/m^3^ at 20 V—owing to its high conductivity. These energy trends directly translated into CO_2_ emissions: for instance, the control emissions were 720, 1440, and 4000 kg/m^3^ for 10 V, 20 V, and oven curing, respectively; 0.75 CF reached 8184 kg/m^3^ at 20 V, while 0.75 EW climbed to 10,848 kg/m^3^, underscoring the linear relationship between curing energy and embodied carbon. Besides energy and CO_2_, the method of curing also significantly impacted water consumption, which, besides being correlated with mixing, was also correlated with evaporation and maintenance during curing. Oven cures needed 560,000 L/m^3^ consistently, but electrical cures at low voltages exhibited a large range—between about 400,000 L/m^3^ and 900,000 L/m^3^ depending on the dosing of fibers—due to highly conductive mixes inducing local Joule heating, encouraging the increased water loss to be compensated for by scaling. High-voltage cures amplified this effect: e.g., for 0.50 EW, it consumed 900,480 L/m^3^ at 10 V but doubled to 1,800,960 L/m^3^ at 20 V; for CB series, also, the water demands rose with dosing, from about 647,640 L/m^3^ at 1% CB and 10 V to almost 1.48 million L/m^3^ at 3% CB and 20 V. These high water amounts directly carried through to soil pollution, eutrophication, and ozone depletion groups because all three are factors of both the upstream raw material production (especially alkali activators and fibers) and the generation of curing using energy. At 10 V, soil pollution ranged between 7315 kg/m^3^ for low CF dosing and 15,437 kg/m^3^ for 0.50 EW, eutrophication between 1219 and 2573 kg PO_4_ eq/m^3^, ozone depletion between 0.122 and 0.257 kg CFC-11 eq/m^3^, and toxicity between 91 and 193 kg DCB eq/m^3^. Doubling the voltage to 20 V approximately doubled each of these values across all series; e.g., for 0.50 EW, at 20 V, it reached 30,874 kg soil eq, 5146 kg PO_4_ eq, 0.515 kg CFC-11 eq, and 386 kg DCB eq—over three times the impacts of oven curing for some categories—showing that fiber conductivity amplifies the environmental impacts of electrical demand. This trend persisted consistently: CF and SF showed intermediate impacts and CB increased impacts with dosing, and EW, despite its mechanical benefits, exhibited the highest environmental footprint due to the high voltage due to its metal composition being sourced through recycling, which allowed for strong current conduction, leading to high current. However, even here, there is a significant circular economy benefit due to its waste source, to some extent compensating for its high operational impacts when looking at its entire life cycle. All of the impact categories were linked by way of the energy pathway. High-voltage fibers increased the current demand, which raised energy consumption, which correspondingly boosted CO_2_ emissions, ozone destruction (through fossil fuel consumption), eutrophication (through the upstream production of activators and fuel), and chemical toxicity for water, soil, and eutrophication (through resource consumption). Water consumption also mediates soil and eutrophication impacts by way of leaching and resource use. On the lower end of the spectrum, electrical curing at 10 V and low to moderate fiber contents (e.g., 0.25–0.50 CF or 0.25 SF) consistently exhibited the minimum impacts across all types, reaching 50–70% below oven curing for all energy and CO_2_ values and also less for the soil, eutrophication, and ozone impacts by similar amounts. The control mixtures cured below 10 V performed comparably well, with the applied voltage exerting the greatest environmental impact among all factors, rather than being directly dependent on the curing method itself. Oven curing, for all its high energy, exhibited moderate water and chemical impacts according to its normalized profile, yet its high-energy characteristic placed it as carbon-intensive. By contrast, 20 V curing—even with highly conductive EW and CB mixes—exhibited the highest overall environmental burden across nearly all categories, indicating that increasing the voltage itself was not beneficial in terms of environmental performance and could even surpass the impacts of oven curing if not carefully optimized.

## 4. Conclusions

This study comprehensively investigated the effect of electrical curing on the physical, mechanical, microstructural, and environmental performance of MK-GBFS-based geopolymer mortars containing different conductive fibers and fillers, and the following conclusions can be drawn from the paper:

(1) Electrical curing efficiency: Rapid electrical curing efficiently converted MK–GBFS geopolymer mortars from laboratory-grade experimental adhesive into field-deployable, high-performance concrete by facilitating accelerated geopolymerization instead of traditional thermal curing.
(2) Comparable strength to thermal curing: Whereas oven curing provided the greatest compressive strength (79.81 MPa for 25MK–75GBFS at 14 M NaOH and 1.25 B/A), the GBFS-heavy mixtures electrically cured at 20 V reached as high as 72.18 MPa, affirming electrical curing’s ability to achieve a strength similar to that under the traditional methods.
(3) Voltage influence: Raising the curing voltages from 10 V to a higher value of 20 V resulted in a considerable strength improvement for all activator molarities and B/A ratios.
(4) Activator concentration: The optimal NaOH contents for electrocuring ranged between 10 M and 14 M, during which proper Na^+^ availability enabled increased the reaction kinetics acceleration.
(5) The binder/activator ratio: Higher B/A ratios (1.25–1.40) resulted in increased compressive strength according to a higher precursor concentration but decreased the workability, suggesting that a compromise is necessary for workable applications.
(6) Role of GBFS: Systems rich in slag had markedly improved strength and conduction compared to those for MK-rich mixes as a result of CaO encouraging more compact C–A–S–H/N–A–S–H gels under electrical fields.
(7) Porosity and water absorption trends: CF and EW fibers increased the water absorption and porosity, especially at higher dosages. CB at 1–2% decreased the porosity through a microfiller effect, whereas 3% CB resulted in agglomeration and build-up of the void content.
(8) The UPVs indicated these porosity variations, displaying a lower velocity for more heavily populated mixes and a higher velocity for low CB contents, evidencing the sensitivity of ultrasonic pulse velocity to matrix compactness.
(9) Abrasion behavior: While the wear resistance decreased with increasing porosity for EW, SF achieved moderate success due to its finer tip and less disruptive network structure.
(10) Mechanical enhancement through fibers: All of the fiber-reinforced concoctions showed increased compressive, bending, and tensile strength when compared to these values for the control. EW, particularly at 0.50%, resulted in the highest compressive strength (88.30 MPa at 7 days), which corresponded to its highest peak current (26.8 A).
(11) Flexural and tensile improvements: SF exhibited significant improvements in flexural and tensile strength, which were caused by their crack-bridging capability and matrix densification.
(12) Dosage optimization: Reasonable dosages (0.50% EW, 0.75% SF, 1% CB) offered the optimum compromise between conductivity and mechanical soundness. High fiber additions resulted in increased porosity and a lower performance for increased current conduction.
(13) The SEM micrographs displayed dense and well-structured matrices in the electrocured samples, while the development of continuous gel phases and a void-reduced microstructure indicated that the microstructure was efficiently enhanced by the electric fields.
(14) The FTIR spectra showed typical Si–O–T bands (≈990–1000 cm^−1^) and C–O bands (1300–1400 cm^−1^), evidencing geopolymerization reactions.
(15) The XRD patterns exhibited broad amorphous humps characteristic of geopolymer gels, as well as crystalline phases of calcite, indicative of both geopolymeric gel precipitation and incidental carbonation.
(16) EDS verified the even distribution of Na, Al, Si, Ca, and Fe in slag-rich systems, favoring the coexistence of N–A–S–H and C–A–S–H gels. Although EDS cannot act as a phase identification tool, its tendency correlated well with the XRD and FT-IR results.
(17) Fiber conductivity’s role: Conductive fibers functioned as percolation routes, enhancing the cure efficiency. Specifically, EW built stable current routes, accelerating the reaction rates and promoting matrix closure densification.
(18) HD method evaluation: Analysis with the HD method, assigning equal weights to each of the parameters, resulted in the election of 0.50% CF, 0.75% SF, 0.75% EW, and 1% CB as the optimum compositions of each of their respective additives. Out of these, the overall best was 0.75% EW, which exceeded the control for 8 of 11 properties, with the porosity parameters being the sole points of relative weakness.
(19) Multi-criteria decision advantage: The HD method struck a good balance in conflicting performance criteria, enabling optimization of the optimum blends, which were not necessarily at the top of the individual categories but provided the optimum overall performance.
(20) The LCA proved 10 V electrical curing to provide the environmentally best performance, cutting the energy demand from 8000 kWh/m^3^ (oven) by as little as 1440 kWh/m^3^ (control) and CO_2_ emissions from 4000 kg/m^3^ to 720 kg/m^3^, by as much as 80%. Of the series, 0.25–0.50 CF and SF at 10 V offered the best balanced profiles for all impact categories, whereas EW at 10 V, despite the increased energy consumption (≈10,800–12,800 kWh/m^3^), scored high for waste valorization. By contrast, 20 V curing for highly conductive mixes like 0.50 EW produced the highest burdens, causing 25,728 kWh/m^3^ of energy use and 12,864 kg CO_2_/m^3^, beating oven curing in a few categories.

With electrical curing accompanied by conductive additives, it is possible to readily produce high-strength geopolymer mortars under field-like conditions, which are a less thermally favored but more operationally appealing substitute for applications. This approach closely aligns with sustainable construction goals, notably diminishing the energy demand and stimulating the conversion of industrial EW into geopolymers and meanwhile promoting circular economy initiatives. By tailoring the porosity-related parameters and setting them equal to all of the LCA studies, it is also feasible to enhance the environmental profile of such systems. In future studies, efforts are recommended to exceed the traditional single- or dual-parameter framework’s capability by employing sophisticated MCDM methodologies like the Analytical Hierarchy Process (AHP) and fuzzy scope methodologies to systematically handle interdependent criteria and data diversity. The AHP permits hierarchical breakdown of complicated decisions and clear-cut quantification of changing attributes’ relative importance, whereas fuzzy scope methodologies are skillful in handling uncertainty, transition states, and fine differences in performance; thus, utilizing such methodologies would help to bring a more adaptable, intelligent, and contextual decision support tool for designing geopolymer blends, which can enhance both the scientific depth and field-practical applicability of follow-up studies performed under field conditions.

## Figures and Tables

**Table 1:** Granulometry of the fine aggregate.

Sieve Size (mm)	Cumulative Retained (%)
2.00	3.72
1.60	9.88
1.00	15.76
0.50	50.10
0.16	95.90
0.08	96.30

**Table 2:** Physical properties of the fibers.

Fiber Type	Diameter (mm)	Length (mm)	l/d Ratio	Electrical Resistivity (Ω·cm)
CF	0.007	12	1714	0.00155
SF	0.4	25	62.5	—
EW	0.4	25	62.5	0.00256

Note: “—” indicates that no electrical resistance measurement was made for the steel fibers.

**Table 3:** Mixing proportions of the geopolymer mortars (units are in grams).

Molarity of NaOH	B/A Ratio	Mix ID	MK	GBFS	Fine Aggregate	Na_2_SiO_3_	NaOH
6 M, 8 M, 10 M, 12 M, 14 M	1.40	100MK0S	450.0	0	1125	210	105
		75MK25S	337.5	112.5	1125		
		50MK50S	225.0	225.0	1125		
		25MK75S	112.5	337.5	1125		
		0MK100S	0	450.0	1125		
	1.25	100MK0S	450.0	0	1125	240	120
		75MK25S	337.5	112.5	1125		
		50MK50S	225.0	225.0	1125		
		25MK75S	112.5	337.5	1125		
		0MK100S	0	450.0	1125		
	1.00	100MK0S	450.0	0	1125	300	150
		75MK25S	337.5	112.5	1125		
		50MK50S	225.0	225.0	1125		
		25MK75S	112.5	337.5	1125		
		0MK100S	0	450.0	1125		

**Table 4:** Bulk density and relative changes in porosity and water absorption of geopolymer mortars.

Mix ID		Bulk Density (kg/m^3^)	Change in Porosity (%)	Change in Water Absorption (%)
Control		1900	-	-
CF	0.25 CF	2020	35.24	27.32
	0.50 CF	2100	70.13	54.22
	0.75 CF	1970	88.99	82.43
SF	0.25 SF	2000	7.49	2.55
	0.50 SF	2060	16.04	7.09
	0.75 SF	2080	44.41	32.30
EW	0.25 EW	2060	31.85	21.68
	0.50 EW	2110	78.77	61.18
	0.75 EW	2140	121.59	97.47
CB	1 CB	2060	−15.86	−22.11
	2 CB	1920	−11.67	−12.30
	3 CB	1800	37.89	45.79

**Table 5:** Results on UPVs.

Mixture		UPV (km/s) (28 Days)	Change in UPV
Control		3.12	-
CF	0.25 CF	2.66	−14.60%
	0.50 CF	3.32	6.50%
	0.75 CF	2.91	−6.84%
SF	0.25 SF	3.21	2.98%
	0.50 SF	3.59	15.11%
	0.75 SF	3.34	6.95%
EW	0.25 EW	3.03	−2.97%
	0.50 EW	3.47	11.24%
	0.75 EW	3.54	13.33%
CB	1 CB	3.08	−1.38%
	2 CB	2.95	−5.56%
	3 CB	2.97	−4.77%

**Table 6:** Atomic percentages of geopolymer samples according to EDS/EDX.

Content	Percentage, %				
	Control	EW	CF	SF	CB
C	42.5	0.2	0.0	0.0	0.0
O	40.1	0.0	0.0	0.0	0.0
Na	3.5	34.3	24.9	31.0	24.5
Al	4.3	45.7	45.5	25.4	35.6
Si	7.2	0.0	0.0	0.0	0.0
Ca	2.5	13.2	13.5	7.8	34.4
Fe	0.0	0.1	0.0	34.4	0.1
Mg	0.0	3.9	12.3	0.4	4.9
K	0.0	2.5	3.8	0.9	0.5

**Table 7:** Results of the HD method.

Main Content	Mixture	K *	The Best Series in the Group	The Best of All
CF	0.25 CF	5.62 × 10^−11^	0.50 CF	0.75 EW
	0.50 CF	6.25 × 10^−16^		
	0.75 CF	1.17 × 10^−15^		
SF	0.25 SF	6.30 × 10^−16^	0.75 SF	
	0.50 SF	5.42 × 10^−16^		
	0.75 SF	1.02 × 10^−16^		
EW	0.25 EW	7.54 × 10^−17^	075 EW	
	0.50 EW	2.51 × 10^−17^		
	0.75 EW	1.74 × 10^−17^		
CB	1 CB	2.34 × 10^−15^	1 CB	
	2 CB	3.97 × 10^−15^		
	3 CB	1.05 × 10^−14^		

* Lower is good.

**Table 8:** The physical and the mechanical properties of control and the best geopolymer mixture.

Mixture	Bulk Density (kg/m^3^)	Porosity, (%)	Water Absorption, (%)	UPV (km/s) @ (28 Days)	Abrasion Resistance cm^3^/50 cm^2^	Comp. Str., 7 Days, MPa	Comp. Str., 28 Days, MPa	Bend. Str., 7 Days, MPa	Bend. Str., 28 Days, MPa	Spl. Tens. Str., 28 Days, MPa	Dir. Tens. Str., 28 Days, MPa
**Control**	1900	9.08	4.77	3.12	16.03	50.8	52.29	9.0	8.9	1.34	1.08
**0.75 EW**	2140 (+)	20.12 (-)	9.42 (-)	3.54 (+)	27.65 (-)	68.86 (+)	76.8 (+)	19.01 (+)	17.04 (+)	2.24 (+)	1.8 (+)

Note: “+” means the superior property, and “-” means the inferior property compared to the reference.

**Table 9:** LCA results for MK–GBFS geopolymer mortars under different curing regimes and reinforcement types.

Series	Curing	Energy (kWh/m^3^)	CO_2_ (kg)	Water (L)	Soil (kg)	Eutro (kg PO_4_ eq)	Ozone (kg CFC-11)	Toxicity (kg DCB)
Control	10 V	1440	720	100,800	1728	288	0.0288	21.6
Control	20 V	2880	1440	201,600	3456	576	0.0576	43.2
Control	Oven	8000	4000	560,000	9600	1600	0.1600	120
0.25 CF	10 V	6096	3048	426,720	7315	1219	0.122	91.4
0.25 CF	20 V	12,192	6096	853,440	14,630	2438	0.244	182.9
0.25 CF	Oven	8000	4000	560,000	9600	1600	0.160	120
0.50 CF	10 V	7164	3582	501,480	8597	1433	0.143	107.5
0.50 CF	20 V	14,328	7164	1,002,960	17,194	2866	0.287	215.0
0.50 CF	Oven	8000	4000	560,000	9600	1600	0.160	120
0.75 CF	10 V	8184	4092	573,000	9821	1637	0.164	122.8
0.75 CF	20 V	16,368	8184	1,146,000	19,642	3274	0.327	245.5
0.75 CF	Oven	8000	4000	560,000	9600	1600	0.160	120
0.25 SF	10 V	7020	3510	491,400	8424	1404	0.140	105.3
0.25 SF	20 V	14,040	7020	982,800	16,848	2808	0.281	210.6
0.25 SF	Oven	8000	4000	560,000	9600	1600	0.160	120.0
0.50 SF	10 V	8220	4110	575,400	9864	1644	0.164	123.3
0.50 SF	20 V	16,440	8220	1,150,800	19,728	3288	0.329	246.6
0.50 SF	Oven	8000	4000	560,000	9600	1600	0.160	120.0
0.75 SF	10 V	9600	4800	672,000	11,520	1920	0.192	144.0
0.75 SF	20 V	19,200	9600	1,344,000	23,040	3840	0.384	288.0
0.75 SF	Oven	8000	4000	560,000	9600	1600	0.160	120.0
0.25 EW	10 V	9072	4536	635,040	10,886	1814	0.181	136.1
0.25 EW	20 V	18,144	9072	1,270,080	21,772	3628	0.363	272.2
0.25 EW	Oven	8000	4000	560,000	9600	1600	0.160	120.0
0.50 EW	10 V	12,864	6432	900,480	15,437	2573	0.257	193.0
0.50 EW	20 V	25,728	12,864	1,800,960	30,874	5146	0.515	386.0
0.50 EW	Oven	8000	4000	560,000	9600	1600	0.160	120.0
0.75 EW	10 V	10,848	5424	759,360	13,018	2170	0.217	162.7
0.75 EW	20 V	21,696	10,848	1,518,720	26,035	4339	0.434	325.4
0.75 EW	Oven	8000	4000	560,000	9600	1600	0.160	120.0
1% CB	10 V	9252	4626	647,640	11,102	1850	0.185	138.8
1% CB	20 V	18,504	9252	1,295,280	22,205	3700	0.370	277.5
1% CB	Oven	8000	4000	560,000	9600	1600	0.160	120.0
2% CB	10 V	9900	4950	693,000	11,880	1980	0.198	148.5
2% CB	20 V	19,800	9900	1,386,000	23,760	3960	0.396	297.0
2% CB	Oven	8000	4000	560,000	9600	1600	0.160	120.0
3% CB	10 V	10,560	5280	739,200	12,672	2112	0.211	158.4
3% CB	20 V	21,120	10,560	1,478,400	25,344	4224	0.422	316.8
3% CB	Oven	8000	4000	560,000	9600	1600	0.160	120.0

## Data Availability

The original contributions presented in this study are included in the article. Further inquiries can be directed to the corresponding author.

## Abbreviations

The following abbreviations are used in this manuscript:

MK	Metakaolin
GBFS	Ground granulated blast furnace slag
SS	Sodium silicate
SH	Sodium hydroxide
SF	Steel fiber
CF	Carbon fiber
CB	Carbon black
EW	Waste erosion wire
UPV	Ultrasonic pulse velocity
SEM	Scanning Electron Microscope
EDS	Energy-Dispersive X-ray Spectroscopy
XRD	X-Ray diffraction
FTIR	Fourier Transform Infrared Spectroscopy

## Appendix A

Holistic evaluation is an evaluation approach in the literature, and decision support methods ease the evaluation of a database of experimental results. In the holistic evaluation approach, independent of the number of parameters, all parameters are used in the calculation steps, and as a result, a constant is usually found. In this way, the constant is used to find the best solution. The HD method is one of the multi-criteria decision support methods used to evaluate alternatives and is used for all parameters. The parameters of other options are presented in the alternatives matrix (Equation (A1)).

(A1) $$M=\begin{array}{c} \left[\begin{array}{ccc} m_{11} & \ldots & m_{1l}\\ \vdots & \ddots & \vdots \\ m_{k1} & \ldots & m_{kl} \end{array}\right] \end{array}$$

where all is the lth criterion of the kth alternative, and M is the score matrix of alternatives.

In this method, if a control is in the database, the parameters of alternatives are divided by the parameter of the control. If a control is not in the database, the mean of the parameters of the alternatives is first determined, and the mean is multiplied by an incrementing constant (i.e., 2, 3). The parameters of the alternatives are divided by the modified/magnified/multiplied mean of the control parameter. Here, it can be recommended that, instead of selecting the mean, the value can be selected as greater than two times the maximum (Equation (A2)).

(A2) $$M_{d}=\begin{array}{c} \left[\begin{array}{ccc} m_{11}/m_{11} & \ldots & m_{1l}/m_{1l}\\ \vdots & \ddots & \vdots \\ m_{k1}/m_{11} & \ldots & m_{kl}/m_{1l} \end{array}\right] \end{array}=\left[\begin{array}{ccc} 1 & \ldots & 1\\ \vdots & \ddots & \vdots \\ d_{k1} & \ldots & d_{kl} \end{array}\right]$$

where M_d_ is the relative criterion matrix of alternatives, and d_kl_ is the lth relative criterion of the kth alternative.

Then, if weighting the parameters is intended, the relative parameters are multiplied by the weights of the parameters (Equation (A3)).

(A3) $$M^{*}=\begin{array}{c} \begin{array}{c} w\\ \vdots \\ w_{n} \end{array}\left[\begin{array}{ccc} m_{11} & \ldots & m_{1l}\\ \vdots & \ddots & \vdots \\ m_{k1} & \ldots & m_{kl} \end{array}\right] \end{array}=\left[\begin{array}{ccc} m_{11}^{*} & \ldots & m_{1l}^{*}\\ \vdots & \ddots & \vdots \\ m_{k1}^{*} & \ldots & m_{kl}^{*} \end{array}\right]$$

where M* is the score matrix of alternatives, and m_kl_ is the lth weighted criterion of the kth alternative.

In the last part, each alternative’s scalar value (K) is calculated using a fraction numerator and denominator function. The fraction numerator and denominator involve multiplying the relative parameters of alternatives by less and greater than 1.0, respectively. The K values are then listed from lower to higher, and the lowest is presented as the best alternative.

The acceptability levels (ALs) of the alternatives can be calculated using the lowest (Klow) and highest (Khigh) values. The difference (ΔK) between the lowest and highest K values is calculated, and the value is divided by 4, representing four regions: the excellent region, the very good region, the good region, and the acceptable region.

With (0 × ΔK + Klow) ≤ K< (0.25 × ΔK + Klow), the alternative is “excellent” compared to the desired/control alternative,

With (0.25 × ΔK + Klow) ≤ K < (0.50 × ΔK + Klow), the alternative is “very good” compared to the desired/control alternative,

With (0.50 × ΔK + Klow) ≤ K < (0.75 × ΔK + Klow), the alternative is “good” compared to the desired/control alternative,

With (0.75 × ΔK + Klow) ≤ K ≤ (1.00 × ΔK + Klow), the alternative is “acceptable” compared to the desired/control alternative.
